# Supplementary material for: Inflammatory factors and risk of lung adenocarcinoma: a Mendelian randomization study mediated by blood metabolites
Source: Front Endocrinol (Lausanne). 2024 Aug 27;15:1446863. doi: 10.3389/fendo.2024.1446863 (PMC11384989; doi:10.3389/fendo.2024.1446863)
Supplement: Supplementary file 1 [file DataSheet1.zip › supplementary files/S2/GCST90200978/forest.pdf]

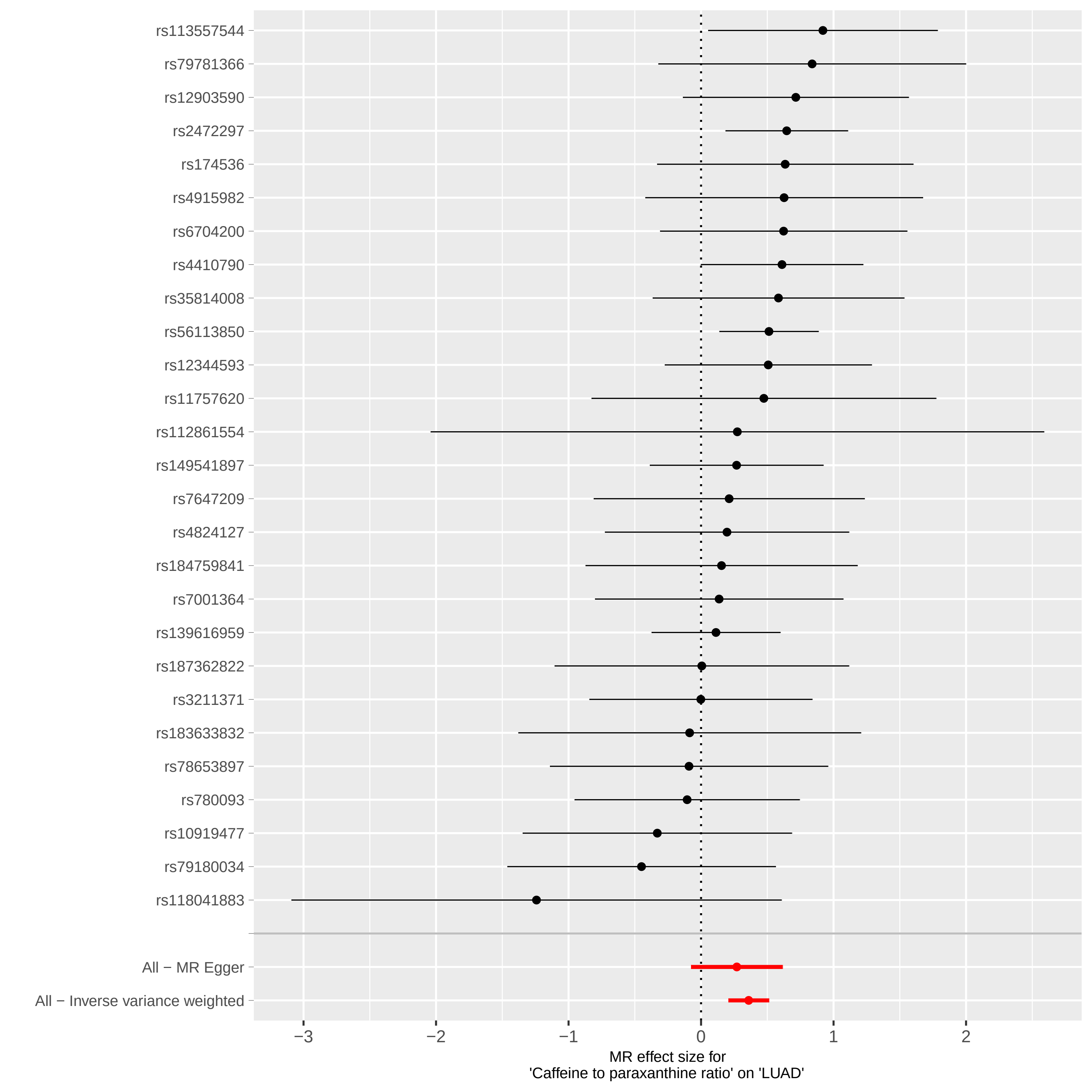
rs113557544
rs79781366
rs12903590
rs2472297
rs174536
rs4915982
rs6704200
rs4410790
rs35814008
rs56113850
rs12344593
rs11757620
rs112861554
rs149541897
rs7647209
rs4824127
rs184759841
rs7001364
rs139616959
rs187362822
rs3211371
rs183633832
rs78653897
rs780093
rs10919477
rs79180034
rs118041883
All − MR Egger
All − Inverse variance weighted
−3
−2
−1
0
1
2
MR effect size for
'Caffeine to paraxanthine ratio' on 'LUAD'
